# Engineered biosynthesis of plant polyketides by type III polyketide synthases in microorganisms

**DOI:** 10.3389/fbioe.2022.1017190

**Published:** 2022-10-14

**Authors:** Chang Liu, Sijin Li

**Affiliations:** Robert F. Smith School of Chemical and Biomolecular Engineering, Cornell University, Ithaca, NY, United States

**Keywords:** type III polyketide synthases, plant polyketides, complete biosynthesis, microorganisms, biosynthesis strategies, biosynthesis achievements

## Abstract

Plant specialized metabolites occupy unique therapeutic niches in human medicine. A large family of plant specialized metabolites, namely plant polyketides, exhibit diverse and remarkable pharmaceutical properties and thereby great biomanufacturing potential. A growing body of studies has focused on plant polyketide synthesis using plant type III polyketide synthases (PKSs), such as flavonoids, stilbenes, benzalacetones, curcuminoids, chromones, acridones, xanthones, and pyrones. Microbial expression of plant type III PKSs and related biosynthetic pathways in workhorse microorganisms, such as *Saccharomyces cerevisiae*, *Escherichia coli*, and *Yarrowia lipolytica*, have led to the complete biosynthesis of multiple plant polyketides, such as flavonoids and stilbenes, from simple carbohydrates using different metabolic engineering approaches. Additionally, advanced biosynthesis techniques led to the biosynthesis of novel and complex plant polyketides synthesized by diversified type III PKSs. This review will summarize efforts in the past 10 years in type III PKS-catalyzed natural product biosynthesis in microorganisms, especially the complete biosynthesis strategies and achievements.

## Introduction

Plant natural products (PNPs, also called specialized metabolites) occupy unique structural space and are of great significance in human medicine, agriculture, and the cosmetic industry. Representative examples include the anticancer agent Taxol (Kingston, 1994), antimalarial drug artemisinin (Klayman, 1985), and analgesic opiates (Pastemak Gavril et al., 1980). Sourcing the valuable PNPs from their native producers relies on plant growth and is usually susceptible to environmental changes. The rapid development of synthetic biology has enabled microbial biomanufacturing as a powerful alternative approach due to microorganisms’ shorter cultivation time and less reliance on climate. Microorganisms are more feasible hosts to engineer than plants due to their simple genetic background and easy genetic operation. Various PNPs have been synthesized in engineered microorganisms in the past decade, including medicinal alkaloids (Galanie et al., 2015; Li et al., 2018b; Srinivasan and Smolke, 2020), terpenes (Belcher et al., 2020), and flavonoids (Shah et al., 2019). This review will focus on the microbial biomanufacturing of another PNP family, namely polyketides, which are valuable PNPs with medicinal, nutraceutical, or agricultural potential.

**FIGURE 1:**
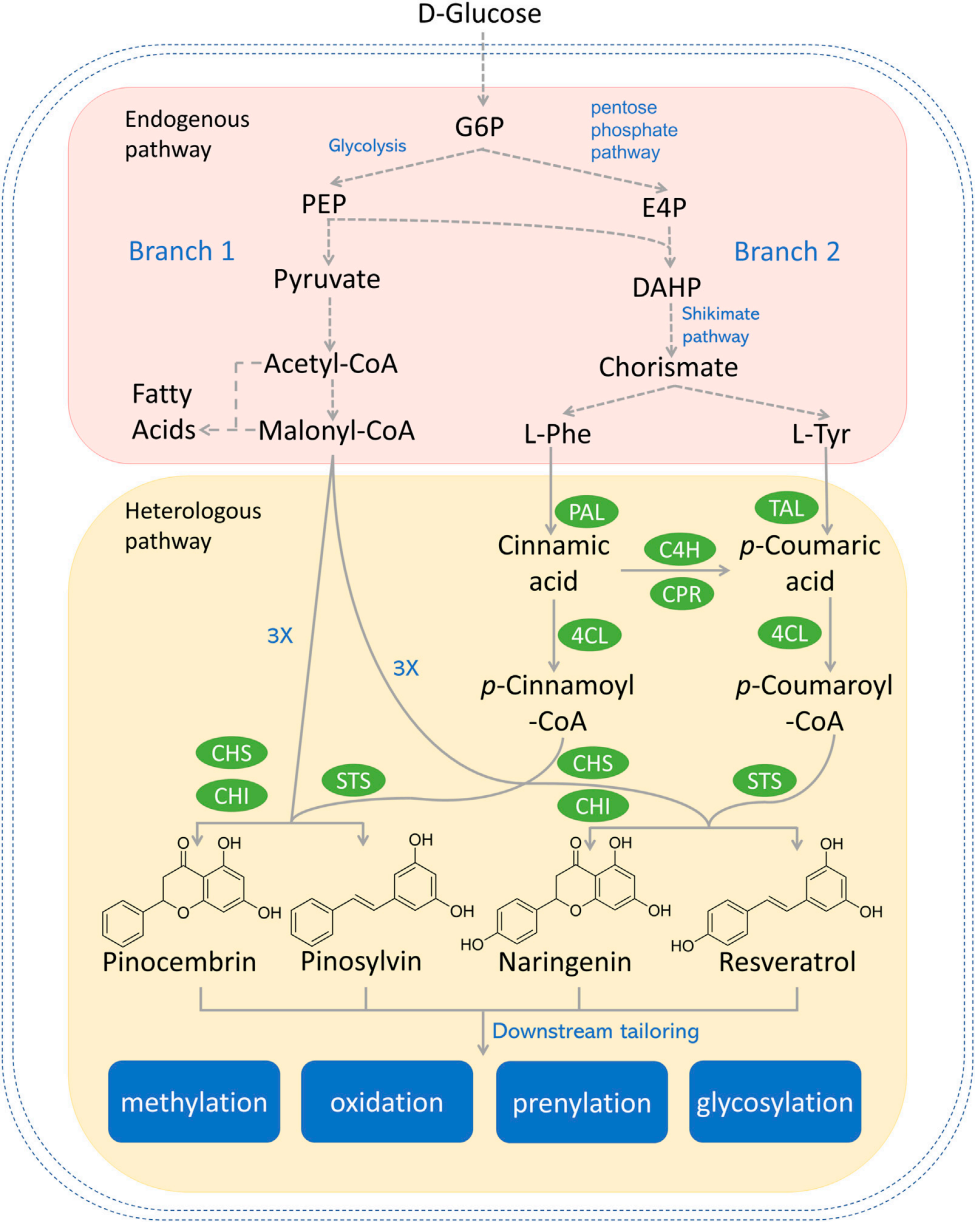
Reconstructed biosynthetic pathway for most explored type III PKS-derived polyketides (e.g., pinocembrin, pinosylvin, naringenin, and resveratrol) in microbial hosts. Dotted arrows refer to multiple steps. Genes and enzymes in green circle are heterologous genes from plants or bacterium. G6P, glucose-6-phosphate; PEP, phosphoenolpyruvate; E4P, erythrose-4-phosphate; DAHP, 3-deoxy-D-arabino-2-heptulosonic acid 7-phosphate; L-Phe, L-phenylalanine; L-Tyr, L-tyrosine; PAL, phenylalanine ammonia lyase; TAL, tyrosine ammonia lyase; C4H, cinnamic acid hydroxylase; CPR, P450 reductase; 4CL, 4-coumaroyl-coA ligase; CHS, chalcone synthase; CHI, chalcone isomerase; STS, stilbene synthase.

Plant polyketides are a large family of PNPs extensively existing in the plant kingdom. The committed reaction in plant polyketide synthesis is a condensation reaction catalyzed by type III polyketide synthases (PKSs) (shown in Figure 1), which condenses a coenzyme A (CoA)-linked starter molecule with several dicarboxyl-CoA extender units (mainly malonyl-CoA) to generate the polyketide scaffold (Dibyendu, 2015). Type III PKSs are mostly found in plants, while type I and type II PKSs are mainly found in bacteria or fungi (Hertweck, 2009). Different types of PKSs vary in both structure and mechanism. Shen (2003) has discussed the differences and provided a detailed diagram of the catalytic mechanisms: Generally, type I PKSs are larger modular and multifunctional enzymes, and each of modules has a different, non-iteratively acting activity that catalyzes one cycle of polyketide chain elongation. Type II PKSs are dissociable complexes and carry out iteratively acting activities. In contrast, type III PKSs are homodimeric proteins that have active sites comprising a Cys, His, Asn catalytic triad in each monomer and carry out iteratively acting condensing activities as well (Morita et al., 2019). The conserved structure of these PKSs allows them to accept various CoA starters and different numbers malonyl-CoAs (Bisht et al., 2021). The substrate preferences of different type III PKSs, along with the downstream tailoring reactions catalyzed by various enzymes (e.g., methyltransferases, oxidoreductases, prenyltransferases, and glycosyltransferases), leads to the large diversity of plant polyketides (Noel et al., 2005). The syntheses of these plant polyketides by type III PKSs also include different catalytic mechanisms, such as C6-C1 Claisen Cyclization and C2-C7 Aldol Cyclization. Morita et al. (2019) discussed the diversity in mechanisms of type III PKSs and gave a hint on how structural subtleties lead to molecular diversities. In this review, we focus on the microbial biosynthesis of plant polyketides from simple sugars by *de novo* pathway reconstruction and engineering, with a particular emphasis on the heterologous expression and engineering of type III PKSs.

## Type III PKS-centered biosynthetic pathway and host strain selection

There are two upstream branch pathways in plants, which lead to the polyketide precursors in the committed step catalyzed by the type III PKS. Similar pathways have been reconstructed in various microorganisms. The graphical summary of the reconstructed type III PKS-centered biosynthetic pathway in microorganisms is shown in Figure 1. Branch 1 starts with D-glucose, which is converted to acetyl-CoA through a series of enzymatic activities. While most acetyl-CoA will enter the tricarboxylic acid (TCA) cycle, a small portion of acetyl-CoA will be converted to malonyl-CoA by the action of acetyl-CoA carboxylase (ACC) (Krivoruchko et al., 2015). Branch 2 starts with aromatic amino acids (AAA, i.e., L-phenylalanine and L-tyrosine), which are deaminated to cinnamic acid or *p*-coumaric acid by the action of monofunctional phenylalanine ammonia lyase (PAL) or bifunctional phenylalanine/tyrosine ammonia lyase (PTAL) in plants (Barros and Dixon, 2020). Alternatively, in reconstructed pathways, L-tyrosine can be deaminated to *p*-coumaric acid catalyzed by tyrosine ammonia lyase (TAL) from bacteria (Jendresen et al., 2015). 4-coumarate-CoA ligase (4CL) conjugates hydroxycinnamic acids to CoAs to form the corresponding CoA esters (e.g., cinnamoyl-CoA and coumaroyl-CoA) (Schneider et al., 2003). Diverse type III PKSs [e.g., chalcone synthase (CHS); stilbene synthase (STS), curcuminoid synthase (CUS)] subsequently condense the starter CoA ester with multiple malonyl-CoAs to form diversified polyketides such as naringenin, pinocembrin, and resveratrol.

Many microbial hosts have been developed for the heterologous production of plant natural polyketides derived from type III PKSs. *Escherichia coli* and *Saccharomyces cerevisiae* are the two most developed hosts due to their unique advantages in biomanufacturing, including fast growth speed and massive genetic manipulation tools that enable rapid pathway construction and optimization (Lian et al., 2018; Pontrelli et al., 2018). As the model bacterial host, *E. coli* can produce a large amount of aromatic amino acids, which can be leveraged for optimal polyketide production (Lütke-Eversloh and Stephanopoulos, 2005; Lütke-Eversloh and Stephanopoulos, 2007). *S. cerevisiae* is a model eukaryotic host particularly suitable for various PNPs’ production. It has similar post-translational modifications as plants to enable functional expression of plant-derived enzymes; the membrane-bound organelles such as endoplasmic reticulum (ER) in yeast provide a similar intracellular environment as in plants for the expression of plant-derived cytochrome P450 enzymes, which are necessary for downstream tailoring of polyketide scaffolds (Huang et al., 2008; Krivoruchko and Nielsen, 2015). Both *E. coli* and *S. cerevisiae* can be engineered to produce naringenin, pinocembrin, and resveratrol from feeding substrates supplemented in the culture medium (e.g., L-phenylalanine and L-tyrosine, cinnamic acid, *p*-coumaric acid, and caffeic acid). Recently, the oleaginous yeast *Yarrowia lipolytica* has emerged as another eukaryotic host to produce plant polyketides due to the high flux through acetyl-CoA precursors, but the genetic operations are still limited by the lack of genetic engineering tools (Abdel-Mawgoud et al., 2018; Markham et al., 2018). More genetic modification strategies are expected in future research. A few non-conventional microorganisms have also been developed to produce plant polyketides, including *Streptomyces venezuelae* and *Corynebacterium glutamicum*. *S. venezuelae* can express large biosynthetic gene clusters and is the common microbial host for the production of complex polyketide production catalyzed by type I and II PKSs (Liu et al., 2018). However, the production of type III PKS-derived polyketides is quite limited. *C. glutamicum* is widely used in amino acid production and has a strong tolerance to aromatic compounds (Becker and Wittmann, 2012; Eberhardt et al., 2014). Engineered *C. glutamicum* can produce naringenin and resveratrol (Kallscheuer et al., 2016; Braga et al., 2018), but endogenous aromatic catabolic pathways must be deleted to inhibit the degradation of aromatic compounds. The key features of each host are summarized in Table 1.

**TABLE 1:** Key features of microbial hosts for polyketide biosynthesis.

	Prokaryotic hosts		Eukaryotic hosts
**Conventional Hosts**	***E. coli***		***S. cerevisiae***
Fast growth speed; massive genetic manipulation tools	Enable extensive aromatic amino acids engineering		Enable functional expression of plant-derived enzymes
**Unconventional Hosts**	***S. venezuelae***	***C. glutamicum***	***Y. lipolytica***
Lack of genetic engineering tools	Enable type I and type II PKS-derived polyketide biosynthesis	Strong tolerance to aromatic compounds	High flux through acetyl-CoA precursors

## Metabolic engineering strategies for polyketides production

One outstanding challenge in plant polyketide biosynthesis is the low production due to the lack of upstream precursors. Feeding expensive upstream precursors is not economical or compatible for larger-scale biomanufacturing. Therefore, engineering the complete plant polyketides biosynthesis pathway from glucose in microbial hosts is important and commercially favorable. The predominant strategy is to increase the availability of both the CoA starter and malonyl-CoA extender. Other strategies include co-culture, flux balance, and gene selection.

### Optimization of the aromatic amino acid pathway

Engineering the shikimate pathway in microbial hosts can increase AAA supply and polyketide production. The shikimate pathway widely exists in plants, algae, fungi, and bacteria. It includes seven enzymatic reactions that combine phosphoenolpyruvate (PEP) and D-erythrose 4-phosphate (E4P) to make chorismate. Chorismate is then converted to AAA, such as L-tyrosine and L-phenylalanine (Rodriguez et al., 2014).

**FIGURE 2:**
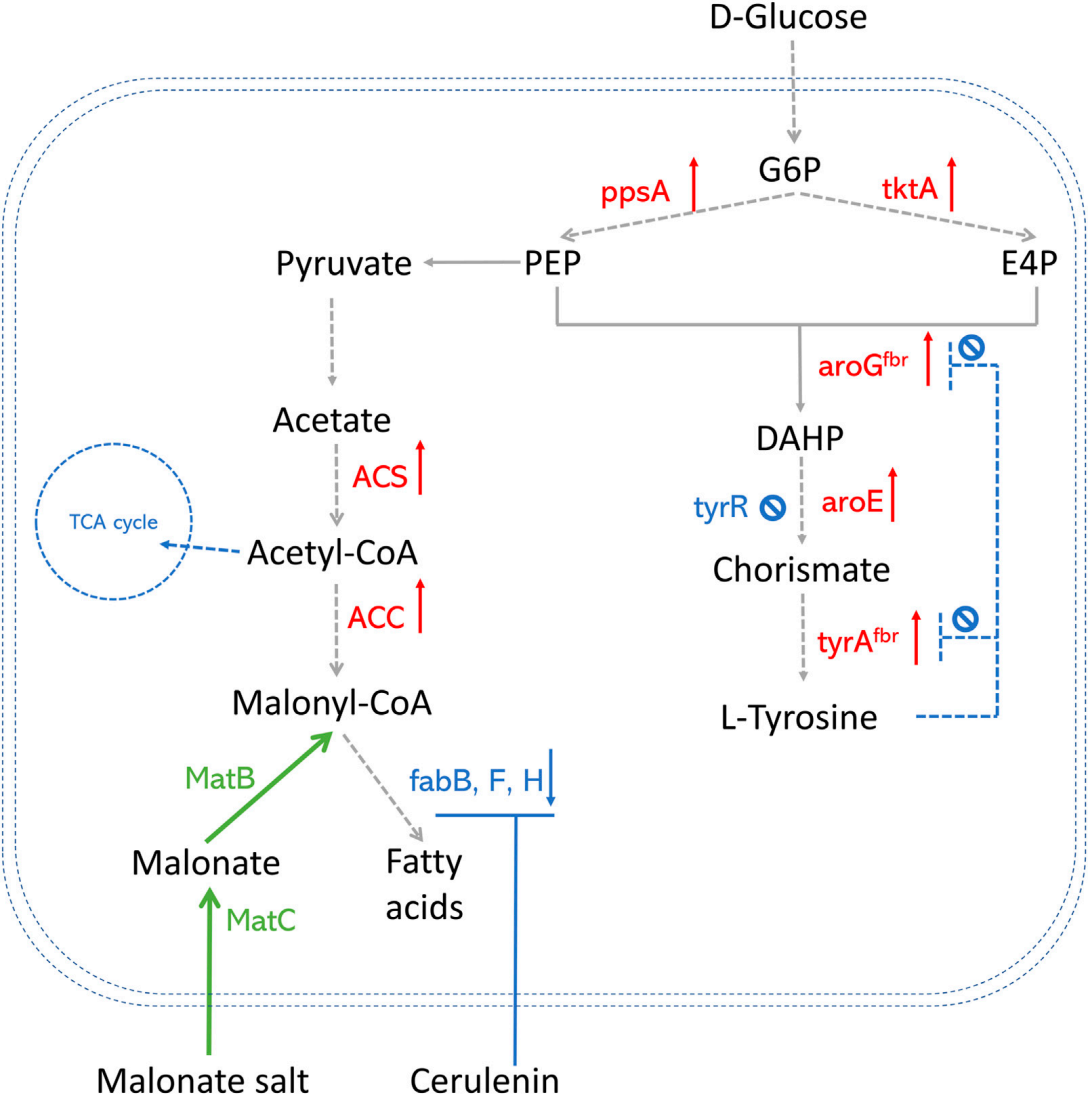
An overview of precursor enhancement engineering in *E. coli.* Genes and enzymes in red are overexpressed. Genes and enzymes in blue are deleted or downregulated. Genes and enzymes in green are heterologous expressed. Dotted arrows refer to multiple steps. G6P, glucose-6-phosphate; PEP, phosphoenolpyruvate; E4P, erythrose-4-phosphate; DAHP, 3-deoxy-D-arabino-2-heptulosonic acid 7-phosphate; fabH, gene that encodes 3-oxoacyl carrier protein synthase III; fabB/fabF, genes that encode the beta-ketoacyl-acp synthase I/II protein; MatB, malonylCoA synthetase; MatC, malonate carrier protein; ACS, acetyl-CoA synthase; ACC, acetyl-CoA carboxylase; TCA cycle, tricarboxylic acid cycle; ppsA, phosphoenolpyruvate synthase; tktA, transketolase; tyrAfbr, chorismate mutase-prephenate dehydrogenase feedback inhibition resistant variant; aroGfbr, DAHP synthase feedback inhibition resistant variant; TyrR, a DNA binding transcriptional regulatory protein.

Successful methods that overproduce AAA in *E. coli* are widely explored (Rodriguez et al., 2014; Jiang and Zhang, 2016). Popular AAA pathway engineering strategies are summarized in Figure 2, including 1) Deleting tyrR, which is a global repressor of AAA biosynthetic pathway and negatively regulates the expression of genes aroF, aroG, aroH, and aroL using AAA as cofactors (Hong et al., 2020; Yuan et al., 2020); 2) Improving PEP and E4P production by overexpressing the phosphoenolpyruvate synthase (ppsA) and transketolase (tktA) genes (Zhang et al., 2017); 3) Alleviating feedback inhibition from L-tyrosine by using 3-deoxy-D-arabino-2-heptulosonic acid 7-phosphate (DAHP) synthase feedback inhibition resistant variant (aroG^fbr^) and chorismate mutase-prephenate dehydrogenase feedback inhibition resistant variant (tyrA^fbr^) (Zhang et al., 2017; Hong et al., 2020; Yuan et al., 2020). Combining several strategies, the *E. coli* strain was able to produce abundant *p*-coumaric acids, which paves the way for polyketide production, especially in co-culture strategies.

**FIGURE 3:**
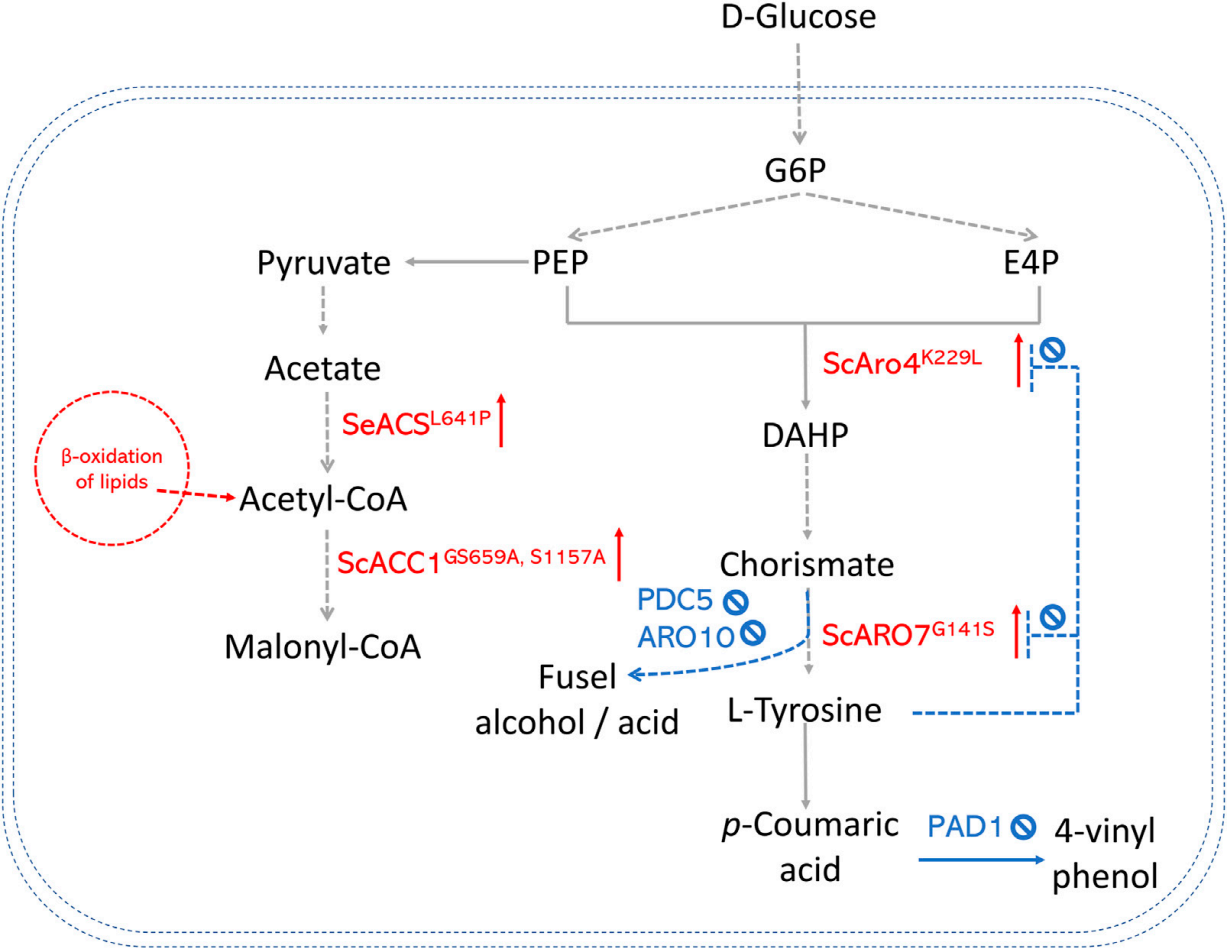
An overview of precursor enhancement engineering in *S. cerevisiae*. Genes and enzymes in red are overexpressed. Genes and enzymes in blue are deleted. Dotted arrows refer to multiple steps. G6P, glucose-6-phosphate; PEP, phosphoenolpyruvate; E4P, erythrose-4-phosphate; DAHP, 3-deoxy-D-arabino-2-heptulosonic acid 7-phosphate; ARO4K229L, DAHP synthase feedback inhibition resistant variants; ARO7G141S, chorismate mutase feedback inhibition resistant variants; PAD1, phenyl acrylic acid decarboxylase; PDC5, pyruvate decarboxylase; ARO10, phenylpyruvate decarboxylase; ACS, acetyl-CoA synthase; ACC, acetyl-CoA carboxylase.

In *S. cerevisiae*, significant efforts have been made to enhance the production of AAA-derived products, as shown in Figure 3. One of the most successful strategies is overexpressing the feedback-insensitive enzyme mutants (i.e., 3-deoxy-D-arabino-heptulosonate-7-phosphate (DAHP) synthase ARO4^K229L^ and chorismate mutase ARO7^G141S^) (Li et al., 2015; Sáez-Sáez et al., 2020). In doing so, the titer of resveratrol in an engineered yeast strain was enhanced by 78% in batch cultures. Downregulating competing branch pathways is also a promising approach. For example, pyruvate decarboxylase (PDC5) and phenylpyruvate decarboxylase (ARO10), which convert hydroxyphenylpyruvate to fusel alcohol or acid, were deleted to direct more metabolic flux to the L-tyrosine production (Li et al., 2021). Combining both strategies, Li et al. (2021) got strains that produced (2S)-naringenin and p-coumaric acid with titers of 43.4 mg/L and 177.9 mg/L. In contrast, the highest titer was only 5.4 mg/L in the strains that only expressed naringenin pathway genes. A new strategy is to increase E4P availability by introducing a phosphoketalose-based pathway into yeast (Liu et al., 2019). Liu et al. (2019) constructed an *S. cerevisiae* strain to produce high levels of *p*-coumaric acid by increasing the E4P availability. They further replaced the promoters of several important genes at key nodes to optimize carbon flux distribution between glycolysis and the AAA biosynthesis pathway. The maximum *p*-coumaric acid titer yielded by the strain was 12.5 g/L. This *p*-coumaric overproducing strain was then used as a background strain to *de novo* biosynthesize bioactive isoflavonoid daidzein with the expression of a CHS (Liu et al., 2021).

### Enhancement of intracellular malonyl-CoA level

The availability of malonyl-CoA in many microbial hosts is limited and considered the rate-limiting step towards diverse polyketides. Multiple strategies have been developed to overcome this bottleneck, summarized in Figures 2, 3. In polyketide-producing microorganisms, malonyl-CoA is usually synthesized from acetyl-CoA catalyzed by acetyl-CoA carboxylases (ACC). Native ACC enzyme in *E. coli* is feedback-inhibited by acyl carrier proteins (ACPs) from the fatty acid biosynthetic pathway (Davis and Cronan, 2001). A widely applied strategy to increase ACC activity is heterologously expressing the ACC enzyme. Zha et al. (2009) enhanced malonyl-CoA titer by 3-fold after introducing *C. glutamicum* ACC into *E. coli*. Wu et al. (2021) found novel ACC families from *Salmonella enterica* that exhibited the highest activities among all the candidates tested by performing a comprehensive phylogenomic and experimental analysis and obtained 1,073.8 mg/L of naringenin production in *E. coli*. Phosphorylation of ACC in *S. cerevisiae* by the sucrose non-fermenting protein 1 (Snf1p) decrease ACC’s activity (Shirra et al., 2001). To address this inhibition, mutant *ScAcc1p* ^*S659A, S1157A*^ was overexpressed to increase ACC activity (Shi et al., 2014). Li et al. (2015) overexpressed this mutated *ScAcc1p* ^*S659A, S1157A*^ in a resveratrol-producing strain and improved resveratrol production from 4.85 mg/L to 6.39 mg/L.

Rewiring the central carbon flux towards malonyl-CoA and malonyl-CoA-derived polyketides is also widely explored (Zha et al., 2009; Lian et al., 2014; Cardenas and da Silva, 2016; Zhang et al., 2019). In *E. coli*, optimization of the glycolytic pathway has increased acetyl-CoA availability and led to 474 mg/L naringenin production (Xu et al., 2011). Deleting the competing acetate and ethanol pathways, inhibiting fatty acid synthesis that consumes malonyl-CoA, and acetyl-CoA synthase (ACS) overexpression resulted in a 15-fold improvement of malonyl-CoA availability and phloroglucinol production of 1,280 mg/L (Zha et al., 2009). Heterologous expression of an ACS mutant from *Salmonella enterica* (*SeACS* ^*L641P*^) with increased activity in *S. cerevisiae* can boost acetyl-CoA availability from acetate (Chen et al., 2013). β-oxidation of fatty acids in yeast peroxisomes, the main pathway for fatty acid degradation, can also be optimized to increase malonyl-CoA supply (Zhang et al., 2021).

Alternatively, in *E. coli*, the intracellular malonyl-CoA level can be elevated by the heterologous expression of the malonate assimilation pathway, including gene *matB* (malonyl-CoA synthetase) and *matC* (malonate carrier protein) from *Rhizobium trifolii* and supplied with malonate at the same time (Wu et al., 2014; Zhou et al., 2019). Moreover, unfavored malonyl-CoA consumption by fatty acid synthesis can be reduced by using cerulenin, a well-characterized chemical inhibitor for fatty acid elongation (Santos et al., 2011; Kallscheuer et al., 2016). The clustered regularly interspaced short palindromic repeats interference (CRISPRi) system was also proposed to identify the candidate genes that can efficiently channel carbon flux toward malonyl-CoA (Wu et al., 2017b; Wu et al., 2021).

### Co-culture strategies

Co-culture of different strains (*E. coli* and *S. cerevisiae*, or different *E. coli* strains) can fully utilize the innate metabolic flux towards AAA and malonyl-CoA and/or reduce the metabolic burden of each strain by dividing extensively long and complicated pathways into individual modules. Various PNPs have been produced by microbial co-culture, including naringenin and resveratrol. *E. coli-E. coli* co-culture systems have enabled the heterologous production of naringenin (Ganesan et al., 2017) and resveratrol (Hong et al., 2020), in which the upstream strain produced *p*-coumaric acid and the downstream strain produced naringenin or resveratrol (55.7 mg/L). An *E. coli-S. cerevisiae* co-culture system produced naringenin from xylose (Zhang et al., 2017). *E. coli* can utilize xylose as the carbon source and excrete acetate, which is then utilized by *S. cerevisiae* as the carbon source (Song et al., 2014) to produce naringenin, leading to 21.16 ± 0.41 mg/L naringenin production. Another E*. coli-S. cerevisiae* co-culture system produced resveratrol from glucose (Yuan et al., 2020), in which an *E. coli* strain produced *p*-coumaric acid, and a downstream *S. cerevisiae* strain produced malonyl-CoA and resveratrol (36 mg/L). In addition to the common polyketides naringenin and resveratrol, some uncommon polyketides and their derivatives have been successfully produced from glucose or feeding substrates (e.g., *p*-coumaric acid or malonate) in microbial hosts through co-culture strategies, including curcuminoids (Fang et al., 2018), anthocyanins (Jones et al., 2017), apigetrin (Thuan et al., 2018), sakuranetin (Wang et al., 2020), and other flavonoids (Du et al., 2020). As the optimal fermentation conditions of each strain in the co-culture (e.g., pH, oxygen, and temperature) are usually different, future improvements in controlling the culture condition dynamically will be helpful to address current challenges in manufacturing. In addition, dynamic tuning of the ratio of different strains during co-culture will be another promising approach to optimize the strategy.

### Gene selection and expression level optimization

There are other bottlenecks hindering the production of plant polyketides. Many studies have found that the conversion of AAAs to polyketides is the limiting step (Santos et al., 2011; Lv et al., 2019). Both enzyme variants and gene expression levels of the essential genes were engineered to address this challenge. For example, the *4CL* gene from *Medicago truncatula* and *CHS* gene from *Vitis vinifera* led to the highest naringenin production among various combinations (Mark et al., 2019). *TALs* and *CHSs* from different organisms can also lead to optimized flavonoid production (Santos et al., 2011; Tong et al., 2022).

Optimizing gene expression levels can also increase polyketide production by increasing gene copy number (enhancing naringenin production in *S. cerevisiae*) (Li et al., 2021), promoter strength, and RBS engineering (enhanced shikimic acid production in *C. glutamicum*) (Zhang et al., 2015), and by tuning gene transcriptional level (enhanced curcumin production) (Kang et al., 2018). Multi-module optimization strategy has been developed to optimize the production of naringenin (Wu et al., 2014) and pinocembrin (Wu et al., 2013): The *de novo* pathway was divided into different modules at the key nodes; Modular expression levels were combinatorially tuned by modifying plasmid gene copy numbers and promoter strengths. Zhou et al. (2019) constructed a library of constitutive promoters in *E. coli* randomly cloned upstream of pathway genes and screened the library to identify a balanced strain with the highest titer. Biosensor-based strategy combined with metabolic circuits was established to dynamically regulate the *de novo* naringenin pathway (Zhou et al., 2021). In this system, the dynamic regulation network contains three layers: the naringenin biosynthesis pathway, *p*-coumaric acid-responsive regulation pathway, and real-time control of intracellular supply of malonyl-CoA. By doing so, 523.7 ± 51.8 mg/L of naringenin was produced. To address the challenge in possible genetic degeneration during long-term cultivation or scale-up, a promising strategy that combines metabolic addiction and negative autoregulation was recently applied in naringenin production in *Y. lipolytica* (Lv et al., 2020). The negative autoregulation using CRISPRi and inducible promoters lowered the competition of malonyl-CoA between the naringenin pathway and the fatty acid pathway; naringenin-responsive transcription factor FdeR coupled naringenin level with leucine synthesis and cell fitness for improved naringenin production. The naringenin-producing population was enriched and 90.9% of naringenin titer still persisted after 324 generations.

## Advanced achievements of plant polyketides in engineered microbial hosts

**FIGURE 4:**
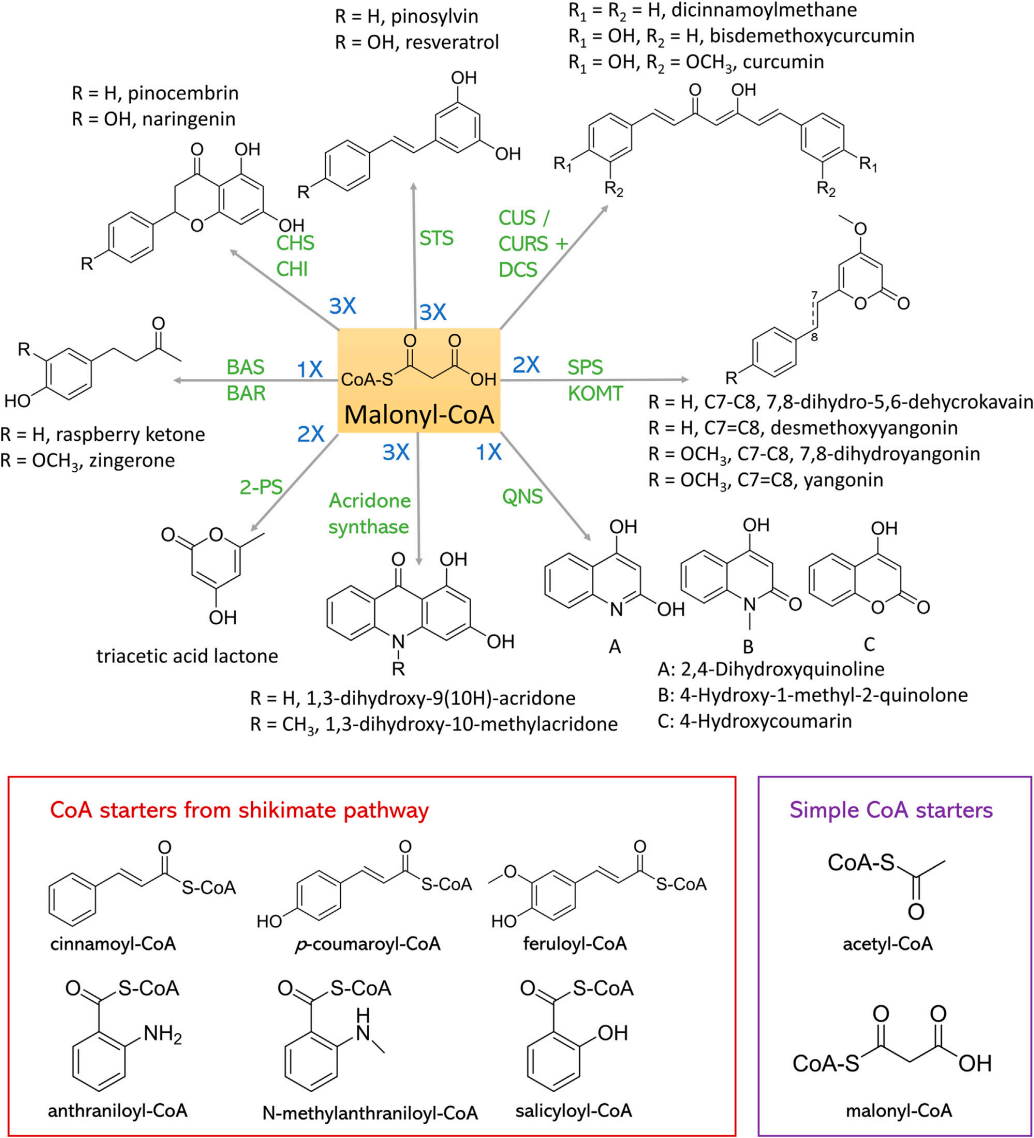
Synthesis of plant polyketides from malonyl-CoA and CoA starters. CHS, chalcone synthase; CHI, chalcone isomerase; STS, stilbene synthase; CUS, curcuminoid synthase; CURS, curcumin synthase; DCS, diketide-CoA synthase; SPS, styrylpyrone synthase; KOMT, kava *O*-methyltransferase; QNS, quinolone synthase; 2-PS, 2-pyrone synthase; BAS, benzalacetone synthase; BAR, benzalacetone reductase.

In this section, we will discuss about recent efforts on the complete synthesis of diverse plant polyketides using different type III PKSs. We will also discuss about a variety of rare polyketides, the complete biosynthetic pathway of which remain yet to be constructed. All these achievements and their strategies are summarized in Table 2 and Figure 4.

**TABLE 2:** Summary of achievements and metabolic engineering strategies for *de novo* production of type III-PKS-derived polyketides.

Microbial host	Product	Essential genes	Optimization strategies	Titer	References
*E. coli*	naringenin	TAL (*R. sphaeroides*), 4CL and CHS (*A. thaliana*)	—	20.8 mg/L	Watts et al. (2004)
*S. cerevisiae*	naringenin/pinocembrin	PAL (*R. toruloides*), 4CL (*A. thaliana*), CHS (*H. androsaemum*)	Δ*PAD1*; *PAL*↑	7.0 mg/L of naringenin, 0.8 mg/L of pinocembrin	Jiang et al. (2005)
*E. coli*	naringenin	TAL (*R. sphaeroides*) 4CL (*S. coelicolor*), CHS (*A. thaliana*), CHI (*P. lobatachi*)	Add cerulenin; control metabolic flux	84 mg/L with cerulenin; 29 mg/L without cerulenin	Santos et al. (2011)
*S. cerevisiae*	naringenin	PAL1, C4H, CPR1, 4CL3, CHS3 and CHI1 (*A. thaliana*), TAL (*R. capsulatus*)	alleviate feedback inhibition: Δ*ARO4*::*ARO4* ^*G226S*^; Δ*ARO3*; eliminate phenylpyruvate decarboxylase: Δ*PDC1*; Δ*PDC5*; Δ*PDC6*	400 μM (shaking flask)	Koopman et al. (2012)
*E. coli*	naringenin	TAL (*R. glutinis*), 4CL (*P. crispum*), CHS (*P. hybrida*), CHI (*Medicago sativa*), matB and matC (*R. trifolii*)	Supply malonate; control flux balance (module engineering strategies by tunning copy numbers and promoter strengths)	100.64 mg/L	Wu et al. (2014)
*E. coli*	naringenin	—	CRISPRi system to identify target genes that increase malonyl-CoA level	421.6 mg/L	Wu et al. (2015)
*E. coli*	naringenin	—	Supply malonate; control flux balance by promoter library	191.9 mg/L	Zhou et al. (2019)
*E. coli*	naringenin	acpH, acpS, acpT, acs, and ACC	Biosensor-based dynamic regulation network control of malonyl-CoA	523.7 mg/L	Zhou et al. (2021)
*S. cerevisiae*	naringenin	TAL (*F. johnsoniaeu*), 4CL (*P. crispum*), CHS (*P. hybrida*), CHI (*M. sativa*)	*ARO4* ^*K229L*^↑ and *ARO7* ^*G141S*^↑; Δ*PDC5* and Δ*ARO10*; multi-copy integration	149.8 mg/L	Li et al. (2021)
*S. cerevisiae*	naringenin	FOX1, FOX2, FOX3, and PEX11	Increase acetyl-CoA supply *via* the *β*-oxidation of fatty acids in the peroxisomes	1,129.44 mg/L (fed batch)	Zhang et al. (2021)
*E. coli*	naringenin	ACC (*S. enterica*)	Use high-activity *ACC* from *S. enterica* and CRSPRi system to regulate malonyl-CoA pathway; dynamic control	1,073.8 mg/L	Wu et al. (2021)
*Y. lipolytica*	naringenin, eriodictyol, taxifolin	CPR (*C. roseus*), F3′H (*G. hybrid*), TAL (*R. toruloides*), CHS and CHI (*P. hybrida*), 4CL and F3H (*S. lycopersicum*)	*CHS*↑ and *CPR*↑; overexpress genes associated with chorismite and malonyl-CoA supply; pH and carbon-nitrogen ratio optimization	252.4 mg/L of naringenin, 134.2 mg/L of eriodictyol, 110.5 mg/L of taxifolin	Lv et al. (2019)
*Y. lipolytica*	naringenin, resveratrol, bisdemethoxycurcumin	4CL (*N. tabacum*), PKS (*H. serrata*), STS (*A. hypogaea*), CUS (*O. sativa*)	*β*-oxidation strategy; *PEX10*↑, *ACC1*↑, *DAHPS* ^*fbr*^ ↑, *SeSam8*↑	898 mg/L of naringenin (fed-batch)	Palmer et al. (2020)
*C. glutamicum*	naringenin, resveratrol	TAL (F. *johnsoniaeu*), STS (*A. hypogaea*), 4CL (*P. crispum*), CHS and CHI (*P. hybrida*)	Add 25 μM cerulenin; delete gene clusters in catabolism of aromatic compounds	32 mg/L of naringenin, 158 mg/L of resveratrol	Kallscheuer et al. (2016)
*S. cerevisiae*	naringenin	CHS (*S. japonica*)	Gene screening for *CHS*	203.49 mg/L (shaking flask)	Tong et al. (2022)
*S. cerevisiae*	naringenin	4CL (*M. truncatula*) and CHS (*V. vinifera*)	Gene screening for *CHS* and *4CL*	28.68 mg/L	Mark et al. (2019)
*E. coli- S. cerevisiae*	naringenin	TAL (*R. toruloides*), 4CL (*P. crispum*), CHS and CHI (*P. hybrid*)	Co-culture strategies; *tktA*↑, *ppsA*↑, *aroG* ^*fbr*^↑, *aroE*↑, *tyrA* ^*fbr*^↑, Δ*pyk*, Δ*pheA*	21.16 mg/L	Zhang et al. (2017)
*E. coli-E. coli*	naringenin	TAL (*R. glutinis*), 4CL (*P. crispum*), CHS (*P. hybrida*), CHI (*Medicago sativa*)	Co-culture strategies	—	Ganesan et al. (2017)
*S. cerevisiae*	naringenin, delphinidin	—	*ARO4* ^*K229L*^↑ and *ARO7* ^*G141S*^ ↑; Δ*PDC5* and Δ*ARO10*; co-culture strategy	144.1 mg/L naringenin	Du et al. (2020)
*S. cerevisiae*	naringenin, kaempferol	—	Gene screening; elimination of competing pathway; optimization the core flavonoid synthetic pathway; co-culture strategy	220 mg/L of naringenin and 200 mg/L of mixed flavonoids; 86 mg/L kaempferol	Lyu et al. (2019)
*E. coli*	pinocembrin	PAL (*R. glutinis*), 4CL (*P. crispum*), CHS (*P. hybrida*), CHI (*M. sativa*), matB and matC (*R. trifolii*)	Supply malonate; control flux balance (module engineering strategies by tunning copy numbers and promoter strengths)	40.02 mg/L	Wu et al. (2013)
*E. coli*	pinocembrin	PAL (*A. thaliana*), 4CL (*O. sativa*), CHS (*P. euramericana*)	Gene screening; overexpress *ACC*	97 mg/L	Kim et al. (2014)
*E. coli*	pinocembrin	PAL (*R. glutinis*), 4CL (*P. crispum*), CHS (*P. hybrida*), CHI (*M. sativa*), matB and matC (*R. trifolii*)	Supply malonate; control flux balance (module engineering strategies by tunning copy numbers and promoter strengths); customize the *PAL* expression level based on modification of the mRNA secondary structure	432.4 mg/L (fed-batch)	Wu et al. (2016a)
*E. coli*	pinocembrin	PAL (*R. glutinis*), 4CL (*P. crispum*), CHS (*P. hybrida*), CHI (*M. sativa*)	CRSPRi system to regulate malonyl-CoA pathway; a two-stage pH control strategy	525.8 mg/L (fed-batch)	Wu et al. (2016b)
*E. coli*	pinocembrin	PAL (*R. mucilaginosa*), 4CL (*S.coelicolor*), CHS (*G. uralensis*), CHI (*M. sativa*)	Engineering fatty acid synthesis	29.9 mg/L	Cao et al. (2016a)
*E. coli*	pinocembrin	PAL (*R. mucilaginosa*), 4CL (*S.coelicolor*), CHS (*G. uralensis*), CHI (*M. sativa*)	Screen gene sources and optimize gene expression levels; *CHS* site-directed mutagenesis; a two-phase pH fermentation strategy	67.82 mg/L	Cao et al. (2016b)
*E. coli*	pinocembrin	—	CRSPRi system to improve ATP level	165.3 mg/L	Tao et al. (2018)
*S. cerevisiae*	pinocembrin derivatives	—	—	10.06 mg/L of chrysin	Liu et al. (2020)
*S. cerevisiae*	resveratrol	TAL (*R. sphaeroides*), 4CL (*A. thaliana*), STS (*V. vinifera*)	Codon optimization and araE transporter	1.06 mg/L	Wang et al. (2011)
*E. coli*	resveratrol and methylated resveratrol analogs	TAL (*S. espanaensis*), 4CL (*S. coelicolor*), STS (*A. hypogaea*), OMT1 and OMT3 (*S. bicolor*)	—	5.2 mg/L of resveratrol	Kang et al. (2014)
*E. coli*	resveratrol	TAL (*R. glutinis*), 4CL (*P. crispum*), STS (*V. vinifera*)	Site-specific integration strategy	4.612 mg/L	Liu et al. (2016)
*E. coli*	resveratrol	TAL (*R. glutinis* or *T. cutaneum*), 4CL (*P. crispum*), STS (*V. vinifera*), matB and matC (*R. trifolii*)	Supply malonate; CRSPRi system to regulate malonyl-CoA pathway; reduce *TAL* mRNA structure	304.5 mg/L	Wu et al. (2017b)
*S. cerevisiae*	resveratrol	TAL (*H. aurantiacus*), 4CL (*A. thaliana*), STS (*V. vinifera*)	*ARO4* ^*K229L*^↑ and *ARO7* ^*G141S*^↑; *ACC1p* ^*S659A, S1157A*^↑; multi-integration	415.63 mg/L from glucose and 531.41 mg/L from ethanol (fed-batch)	Li et al. (2015)
*S. cerevisiae*	resveratrol, pinostilbene and pterostilbene	PAL, C4H and 4CL (*A. thaliana*), STS (*V. vinifera*), ROMT (*V. vinifera* or *S. bicolor*)	Enhance P450 activity; increase copy number of resveratrol pathway genes; *ARO4* ^*K229L*^↑ and *ARO7* ^*G141S*^↑; *ACC1p* ^*S659A, S1157A*^↑; Δ*ARO10*; *EcaroL*↑; *SeACS* ^*L641P*^↑	800 mg/L of resveratrol (fed-batch); 5.52 ± 2.84 mg/L of pinostilbene and 34.93 ± 8.53 mg/L of pterostilbene (feed-in-time medium)	Li et al. (2016)
*S. cerevisiae*	resveratrol	PAL, C4H and 4CL (*A. thaliana*), STS (*V. vinifera*), ROMT (*V. vinifera* or *S. bicolor*)	—	187.07 ± 19.88 mg/L	Costa et al. (2021)
*C. glutamicum*	resveratrol	TAL (*F. johnsoniaeu*), STS (*A. hypogaea*), 4CL (*P. crispum*)	Fermentation strategies	12 mg/L (batch), 59 mg/L when adding cerulenin	Braga et al. (2018)
*Y. lipolytica*	resveratrol	TAL (*H. aurantiacus or* (*F. johnsoniaeu*), 4CL (*A. thaliana*), STS (*V. vinifera*)	*ScAR O 4* ^*K229L*^↑and *ScAR O 7* ^*G141S*^↑or *YlAR O 4* ^*K221L*^↑and *YlAR O 7* ^*G139S*^↑; Δ*ARO10* and Δ*PDC5*; *YlACC1* ^*S667A, S1178A*^↑; overexpress pathway genes	12.4 ± 0.3 g/L (fed-batch)	Sáez-Sáez et al. (2020)
*E. coli-E. coli*	resveratrol	TAL (*R. glutinis*), 4CL (*P. crispum*), STS (*A. hypogaea*)	Co-culture strategies; strain 1: *aroG* ^*fbr*^↑and *tyrA* ^*fbr*^↑; Δ*pgi* and Δ*tyrR*; *TAL*↑; strain 2: Δ*zwf*; *ACC*↑	55.7 mg/L	Hong et al. (2020)
*E. coli -S. cerevisiae*	resveratrol	TAL (*T. cutaneum*), 4CL (*A. thaliana*), STS (*V. vinifera*)	Co-culture strategies; strain 1: *aroG* ^*fbr*^↑ and *tyrA* ^*fbr*^↑; Δ*tyrR*; strain 2: *ACC1p* ^*S659A, S1157A*^↑	36 mg/L	Yuan et al. (2020)
*E. coli-E. coli*	resveratrol	TAL (*P. chrysosporium*), 4CL (*A. thaliana*), STS (*V. vinifera*)	Co-culture strategies; CRSPRi system to regulate malonyl-CoA pathway; develop a resveratrol circuit	204.8 mg/L	Li et al. (2022)
*E. coli*	pinosylvin	PAL (*P. crispum* or *A. thaliana*), 4CL (*S. coelicolor* or *A. thaliana*), STS (*P. densiflora* or *P. strobus*)	Add cerulenin; *in vivo* evolution of *STS*	70 mg/L	van Summeren-Wesenhagen and Marienhagen, (2015)
*E. coli*	pinosylvin	PAL (*R. glutinis or T. cutaneum*), 4CL (*A. thaliana*), STS (*V. vinifera*)	Change 5′ region of mRNA secondary structure; CRSPRi system to regulate malonyl-CoA pathway; flux balance	281 mg/L	Wu et al. (2017a)
*E. coli*	Dicinnamoyl-methane and bisdemethoxycurcumin	PAL (*A. thaliana*), TAL (*S. espanaensis*), 4CL and CUS (*O. sativa*)	Shikimate pathway engineering	6.95 mg/L of dicinnamoylmethane and 4.63 mg/L of bisdemethoxycurcumin	Kim et al. (2017)
*E. coli*	curcumin	DCS and CURS (*C. longa*)	Control flux balance	3.9 ± 0.8 mg/L	Kang et al. (2018)
*E. coli-E. coli*	bisdemethoxycurcumin	TAL (*R. glutinis*), 4CL (*A. thaliana*), CUS (*O. sativa*)	Co-culture strategies; increase malonyl-CoA level: supply malonate	6.28 mg/L	Fang et al. (2018)
*S. cerevisiae*	raspberry ketone	PAL (*R. toruloides*); C4H (*A. thaliana*), 4CL (*A. thaliana* or *P. crispum*), BAS (*R. palmatum*)	Fusion protein of 4CL and BAS	2.8 mg/L or 3.5 mg/L (aerobic fermentation)	Lee et al. (20016)
*E. coli*	raspberry ketone	TAL (*R. glutinis*), 4CL (*A. thaliana*), BAS (*R. rubrum*)	Control flux balance; supply malonate	12.9 mg/L	Moore et al. (2021)
*E. coli*	zingerone	PmPKS (*Piper methysticum*)	Overproduce tyrosine	24.03 ± 2.53 mg/L	Heo et al. (2021a)
*S. cerevisiae*	stytylpyrones	PAL and 4CL (*A. thaliana*), TAL (*R. sphaeroides*), SPS and KOMT (*Piper methysticum*)	*ARO4* ^*Q166K*^↑ and *ARO7* ^*T226I*^↑; overexpress pathway genes by CRISPR-based δ-integration	4.40 μM of 7,8-dihydro-5,6-dehydrokavain, 1.28 μM of desmethoxyyangonin and 0.10 μM of yangonin	Wu et al. (2022)
*E. coli*	stytylpyrones	PnPKS (*Piper nigrum*)	Overproduce tyrosine	52.8 mg/L of 11-methoxy-bisnoryangonin	Heo et al. (2021b)
*E. coli*	acridone derivatives	ACS and NMT (*R. graveolens*); badA (*R. palustris*); pqsA (*P. aeruginosa*)	Shikimate pathway engineering; *ACC*↑	17.3 mg/L DHA and 26.0 mg/L NMA	Choi et al. (2020)
*E. coli*	quinolone derivatives	pqsA and pasD (*P. aeruginosa*); NMT and ACS (*R. graveolens*); QNS (*C. macrocarpa*)	Shikimate pathway engineering; *ACC*↑	255.4 mg/L 4-HC, 753.7 mg/L DHQ, and 17.5 mg/L NMQ	Choo and Ahn, (2019)

### Chalcone synthase

Chalcone Synthase (CHS) is one of the most explored type III PKSs, essential in flavonoid chalcone synthesis. Various chalcones are important intermediates for flavonoid synthesis in plants. The CHS is ubiquitous in almost any plant species (Rammohan et al., 2020). CHS uses C6-C1 Claisen cyclization to produce naringenin chalcone, pinocembrin chalcone, and eriodictyol chalcone from *p*-coumaroyl-CoA, cinnamoyl-CoA, and caffeoyl-CoA, respectively, with three extender malonyl-CoAs. These flavonoid chalcones can further convert to corresponding flavonoids *via* spontaneous reaction or catalyzed by chalcone isomerase (CHI). CHS could also produce isoliquiritigenin with chalcone reductase (CHR) in a concerted manner. Given the antiviral, antioxidant, antitumor, and anti-inflammatory effects of plant flavonoids and their high safety profile (Dao et al., 2011), these CHS-derived flavonoids have very high medical or nutritional value. Therefore, the demand for flavonoid biosynthesis in microorganisms is increasing rapidly. Numerous achievements have been accomplished in recent years, paving the way for industrial biomanufacturing.

The first *CHS* gene sequence was reported by Reimold et al. (1983) in 1983 from cultured parsley cells (*Petroselinum Hortense*). Later, more than 650 *CHS-like* gene sequences were identified in various plants (Bisht et al., 2021). The first CHS structure was elucidated from alfalfa CHS in 1999, revealing its specific homodimeric structure (Ferrer et al., 1999). CHSs combined with 4CLs and CHIs are widely used in chalcone biosynthesis. To achieve *de novo* naringenin chalcone and naringenin biosynthesis in microbial hosts, TAL is introduced to convert L-tyrosine to *p*-coumaric acid, which is the precursor of naringenin chalcone. Naringenin was firstly biosynthesized from simple carbon source glucose in *E. coli* (Watts et al., 2004) and *S. cerevisiae* (Jiang et al., 2005) decades ago, though with a quite low titer. In the following years, numerous strategies were applied to increase the production, such as increasing essential precursors malonyl-CoA (Wu et al., 2014; Wu et al., 2015; Zhou et al., 2019; Wu et al., 2021; Zhang et al., 2021) and L-tyrosine or L-phylalanine (Koopman et al., 2012; Li et al., 2021), screening and overexpressing essential genes (Lv et al., 2019; Mark et al., 2019; Tong et al., 2022), and co-culture strategies (Ganesan et al., 2017; Zhang et al., 2017). Some major achievements are summarized in Table 2. Till now, the highest naringenin production from glucose reported is 1,129.44 mg/L in yeast fed-batch fermentation (Zhang et al., 2021). All these efforts combined to build a solid and stable naringenin-producing platform. Naringenin biosynthesis pathway in microbial hosts especially *E. coli* and *S. cerevisiae*, have been widely used to develop novel synthetic biology tools, such as malonyl-CoA biosensor development (Yang et al., 2018), evolution-guided optimization (Raman et al., 2014). Strategies developed in naringenin *de novo* biosynthesis laid a solid foundation for other valuable plant natural polyketides biosynthesis in microbial hosts. Pinocembrin, another plant polyketide derived from CHS, can serve as a good example. PAL was introduced in pinocembrin-producing strains to convert L-phenylalanine to cinnamic acid. Similar strategies, such as ACC overexpression (Kim et al., 2014) and flux balance control (Wu et al., 2013; Wu et al., 2016a), were applied to enhance pinocembrin titers. Knowing the pH impact on different stages of fermentation, a two-stage pH strategy was explored (Cao et al., 2016a; Wu et al., 2016b). Tao et al., 2018 used the CRISPRi system to identify genes regulating ATP levels and increased pinocembrin titer to 165.3 mg/L.

The *de novo* naringenin and pinocembrin pathways were further engineered to produce numerous medically important derivatives from glucose. The bioactive isoflavonoids genistein and daidzein are downstream products of naringenin and liquiritigenin catalyzed by isoflavone synthase (IFS), which can serve as good examples. Liu et al. (2019) used a *p*-coumaric acid overproducing yeast strain to produce isoflavonoids (Liu et al., 2021). Chalcone reductase (CHR), IFS, and 2-hydroxyisoflavanone dehydratase (HID) were co-expressed with the naringenin biosynthesis pathway. By enhancing the activity of cytochrome P450, which are monooxygenases (i.e., cinnamic acid hydroxylase C4H and IFS), and improving metabolic flux, up to 85.4 mg/L daidzein was produced. Kaempferol, which has positive effects on cancer cell regulation (Chen and Chen, 2013), is a downstream product of naringenin *via* the catalysis of flavanone 3-hydroxylase (F3H) and flavonol synthase (FLS) enzymes. Lyu et al. (2019) optimized kaempferol production to 86 mg/L by gene screening, core flavonoid synthetic pathway optimization, and relocalization into mitochondria of F3H and FLS. Combined with co-culture strategies, 220 mg/L of naringenin and 200 mg/L of mixed flavonoids were obtained. Other examples are delphinidin (Du et al., 2020), ponciretin and sakuranetin (Kim et al., 2013), and anthocyanin (Levisson et al., 2018), all of which are naringenin derivatives in plants. Though *de novo* biosynthesis has not been reported yet, some derivative compounds can be synthesized in microbial hosts by adding precursors. For example, prenylated naringenin can be synthesized in yeast by using prenyltransferases (PTs) from bacteria and adding L-phenylalanine and Dimethylallyl pyrophosphate (DMAPP) (Isogai et al., 2021). With the developed *de novo* metabolic engineering strategies, the complete biosynthesis of these compounds can be realized in the future.

### Stilbene synthase

Stilbene is another most studied plant natural polyketide, which is biosynthesized by a type III PKS stilbene synthase (STS). STS shares 75%–90% sequence similarity with CHS but only exists in a few species, such as *Vitaceae, Gnetaceae, Dipterocarpaceae, Pinaceae, Poaceae, Fabaceae, Leguminosae,* and *Cyperaceae* families (Austin et al., 2004). The catalytic mechanisms of STS and CHS are also different. STS enables iterative decarboxylative condensation of three molecules of malonyl-CoA with one molecule of *p*-coumaroyl-CoA or cinnamoyl-CoA following a C2-C7 aldol cyclization to produce resveratrol and pinosylvin respectively.

Resveratrol is medically valuable to many diseases, such as diabetes, cardiovascular diseases, and neurological disorders (Bhatt et al., 2012; Tomé-Carneiro et al., 2012; Turner et al., 2015) and is also an important additive in foods and cosmetics. *De novo* biosynthesis of resveratrol in microbial hosts has been explored for over a decade with numerous achievements, listed in Table 2. Complete resveratrol biosynthesis was achieved in *S. cerevisiae* for supplementing extra resveratrol in white wine, in which codon-optimized *TAL* mutant and *araE* transporter were used to improve resveratrol production (1.9 mg/L when supplied with tyrosine, and 1.06 mg/L without supplementation) (Wang et al., 2011). An *E. coli* strain was developed to produce resveratrol, and methylated resveratrol that has better bioavailability from glucose (Kang et al., 2014), resulting in a production of 5.2 mg/L resveratrol and four distinct methylated resveratrol analogues were produced by the action of a series O-methyltransferase genes from sorghum. A site-specific integration strategy led to 4.61 mg/L resveratrol from glucose in *E. coli* (Liu et al., 2016). Li et al. (2015) used a stepwise engineering strategy to produce resveratrol, which led to 415.65 mg/L or 531.41 mg/L resveratrol from glucose or ethanol, respectively. The same group also constructed another yeast strain to produce resveratrol from the phenylalanine pathway rather than the tyrosine pathway (Li et al., 2016). After enhancing P450 activity, overexpressing pathway genes, and increasing the precursor supply, a final production of 800 mg/L was achieved in fed-batch fermentation from glucose. In 2021, Costa et al. (2021) used a mixed medium with 2% glucose and 5% ethanol to produce resveratrol and achieved a maximum 187.07 ± 19.88 mg/L production. Multiple similar strategies used in naringenin production were also applied in resveratrol production in microbial hosts to obtain higher production (Wu et al., 2017b; Hong et al., 2020; Sáez-Sáez et al., 2020; Yuan et al., 2020; Li et al., 2022).

Pinosylvin has important medical functions in treating various cancers and cardiovascular, inflammatory diseases (Jeong et al., 2013; Park et al., 2013). van Summeren-Wesenhagen and Marienhagen (2015) increased pinosylvin production by adding cerulenin to increase intracellular malonyl-CoA level and *in vivo* evolving stilbene synthase from *Pinus strobus* for higher activity. 70 mg/L pinosylvin from glucose was achieved. Muti-module optimization strategy combined with malonyl-CoA availability enhancement strategy, 281 mg/L pinosylvin was obtained from glucose (Wu et al., 2017a). Xu et al. used pinosylvin biosynthesis pathway to explore the correlation between the acylation level of proteins and the titer of malonyl-CoA-derived metabolites and found oversupply of malonyl-CoA leads to malonylation of important enzymes involved in biosynthetic pathway, thus inhibiting pinosylvin productivity (Xu et al., 2018).

### Curcuminoid synthase

Curcuminoids are highly produced in the spice turmeric and possess many bioactivities, including anti-inflammatory (Aggarwal and Sung, 2009), anticancer (Wilken et al., 2011), antioxidant (Jayaprakasha et al., 2006) activities. Curcuminoids synthesized in *Curcuma longa* require the actions of two type III PKSs, namely diketide-CoA synthase (DCS) and curcumin synthase (CURS) to form the C6-C7-C6 core structure (Katsuyama et al., 2009). DCS condenses one *p*-coumaroyl-CoA or feruloyl-CoA with one malonyl-CoA to synthesize *p*-coumaroyl-diketide-CoA and feruloyl-diketide-CoA, which will be further condensed with another *p*-coumaroyl-CoA or feruloyl-CoA to form bisdemethoxycurcumin or curcumin by the action of CURS, respectively. Meanwhile, curcuminoids synthesized in *Oryza sativa* require only one type III PKS, named curcuminoid synthase (CUS). By employing similar three-step reaction, bisdemethoxycurcumin can be synthesized from two molecules of p-coumaroyl-CoA and one molecule of malonyl-CoA (Katsuyama et al., 2009). When using cinnamoyl-CoA or feruloyl-CoA as the substrate, dicinnamoylmethane or curcumin can be produced, respectively.

Co-expression of ClDCS with ClCURS or OsCUS in microbial hosts have produced a variety of curcuminoids. The highest curcumin production (563.4 mg/L) was achieved in *E. coli* when ferulic acid was fed as the substrate (Rodrigues et al., 2020). However, as a more complex polyketide, the titer of *de novo* production is much lower. Engineered *E. coli* strains with TAL, PAL, 4CL, and OsCUS and optimized shikimate pathway can synthesize two curcuminoids, dicinnamoylmethane and bisdemethoxycurcumin, from glucose (Kim et al., 2017), but the productions are only 4.63 mg/L (bisdemethoxycurcumin) and 6.95 mg/L (dicinnamoylmethane). Multiplex automatic genome engineering (MAGE) was used to construct a library of 5′- UTR sequences for curcumin pathway optimization, which improved curcurmin production by 38.2-fold compared to the parental strain to 3.9 ± 0.8 mg/L (Kang et al., 2018). *E. coli-E. coli* co-culture system produced bisdemethoxycurcumin from glucose (Fang et al., 2018), in which an *E. coli* strain with 4CL and CUS produced bisdemethoxycurcumin from p-coumaric acid, and the other strain harboring TAL produces p-coumaric acid from glucose. Malonyl-CoA level was also improved by introducing MatB and MatC to transform supplemented malonate directly into malonyl-CoA. Finally, 6.28 mg/L bisdemethoxycurcumin was produced from glucose when malonate was supplied (Fang et al., 2018).

### Benzalacetone synthase

BAS shares 70% amino acid sequence identity with CHS. It condenses one *p*-coumaroyl-CoA with one malonyl-CoA to produce C6-C4 diketide benzalacetone, which can lead to valuable flavoring agent such as raspberry ketone used in cosmetics, food industry, and pharmaceutics (Wang et al., 2012; Bredsdorff et al., 2015; Kim et al., 2016). Till now, BAS have been cloned from *Rheum palmatum, Wachendorfia thyrsiflora, Rubus idaeus*, and *Polygonum cuspidatum* (Shimokawa et al., 2012).

Plant-derived and chemical synthesized raspberry ketone are expensive. Therefore, synthesizing this compound *via* synthetic biology tools in microbial hosts is appealing. A lot of efforts have been made in producing raspberry ketone from *p*-coumaric acid or tyrosine (Wang et al., 2019; Milke et al., 2020; Chang et al., 2021; Yang et al., 2021). *De novo* production has been recently achieved by the co-expression of four heterologous genes (PAL/TAL, C4H, 4CL, and BAS) in an *S. cerevisiae* strain (Lee et al., 2016). Fusion protein of 4CL and BAS further increased the titer by five-fold to 7.5 mg/L from 3 mM *p*-coumaric acid, or 2.8 mg/L *de novo*. A similar five-step raspberry ketone-producing pathway was reconstructed in *E. coli* (Moore et al., 2021), and led to 12.9 mg/L raspberry ketone production by fine-tuning the expression level of each pathway gene using a promoter library.

Zingerone is another phenylbutanones and the key component responsible for the pungency of ginger. Zingerone has various medical importance, including anti-inflammatory, antioxidant, antihyperlipidemic, anticancer, and antibacterial activities (Ahmad et al., 2015). The biosynthetic pathway of zingerone is similar with that of raspberry ketone, while zingerone starts with ferulic acid as the precursor and raspberry ketone biosynthesis starts with p-coumaric acid. Recently, a type III PKS gene (*PmPKS*) from *Piper methysticum* (kava) which exhibits feruloyl-CoA-preferred BAS activity has been identified (Heo et al., 2021a). Consequently, an *E. coli* strain harboring 4CL, PmPKS and benzalacetone reductase (BAR) produced zingerone from ferulic acid, which led to a *de novo* zingerone pathway assembled in an L-tyrosine-overproducing *E. coli*, that produced 24.03 ± 2.53 mg/L zingerone.

### Styrylpyrone synthase

Plant stytylpyrones are mainly found in angiosperm plants, including *Piperaceae, Lauraceae, Annonaceae, Ranuculaceae* and *Zingiberaceae* (Beckert et al., 1997). They usually serve as defense role when attacked by bacterium or fungi, and exhibit anxiolytic, analgesic, or neuroprotective effects (Tzeng and Lee, 2015). Plant styrylpyrone scaffold is formed by C5-C1 Claisen cyclization condensation of one hydroxycinnamoyl-CoA with two malonyl-CoA catalyzed by SPS (Pluskal et al., 2019).

Kavalactones are methylated stytylpyrones derived from kava, which have well-established anxiolytic and analgesic properties and interact with the human central nervous system through mechanisms distinct from those of conventional psychiatric drugs (Chua et al., 2016). In 2019, Pluskal et al. (2019) characterized two *CHS-like* genes encode functional SPSs from *P. methysticum*, namely *PmSPS1 and PmSPS2*. Complete biosynthesis of plant styrylpyrones was achieved in yeast (Wu et al., 2022). Firstly, a styrylpyrone-producing strain containing PmSPS1 and kava *O*-methyltransferase 1 (PmKOMT1) enable diverse plant styrylpyrones production from fed substrates (e.g., *p*-coumaric acid and cinnamic acid). Then the rate-limiting steps were identified and optimized by enzyme overexpression *via* yeast genomic integration. To further enhance the production, a CRISPR-based δ-integration method was used to overexpress the codon-optimized yPmSPS and yPmKOMT1 in yeast. Finally, the strains produced various plant stytylpyrones *de novo*, with the titers of 7,8-dihydro-5,6-dehydrokavain, desmethoxyyangonin and yangonin at 4.40, 1.28, and 0.10 μM, respectively. In another example, an SPS-like enzyme, PnPKS, was recently found from the leaves of black pepper (*Piper nigrum*), catalyzing two-chain elongation with feruloyl CoA-linked starter substrates to form a styrylpyrone 11-methoxy-bisnoryangonin (Heo et al., 2021b). After characterizing the SPS function with feruloyl-CoA of PnPKS, an artificial pathway that can produce 11-methoxy-bisnoryangonin was introduced into an engineered L-tyrosine overproducing *E. coli* strain, resulting in complete production with a titer up to 52.8 mg/L. These studies laid the foundation for industrial scale styrylpyrone biosynthesis and the complete biosynthesis of more complicated plant styrylpyrones from simple carbon source.

### Acridone synthase and quinolone synthase

Both acridone synthase and QNS are from *Rutaceae* plant family, the products of which lead to anthranilic acid-derived alkaloids including acridone alkaloids and quinolone alkaloids. These PNPs exhibit numerous pharmaceutical potentials including anticancer, antimicrobial, antimalarial, antipsoriatic activities (Whitlon et al., 1978; Foley and Tilley, 1998; Michael, 2003; Goodell et al., 2006; Putic et al., 2010). Acridone synthase and QNS mainly use *N*-methylanthraniloyl-CoA instead of p-coumaroyl-CoA as starter substrate. Despite the structural similarities, acridone synthase and QNS catalyze different reactions. Acridone synthase performs the C6-C1 Claisen cyclization of one *N*-methylanthraniloyl-CoA and three malonyl-CoA units to produce 1,3-dihydroxy-N-methylacridone, a common intermediate which can form more complex acridones such as rutacridone. QNS catalyzes the decarboxylation condensation of starter one *N*-methylanthraniloyl-CoA and one malonyl-CoA which spontaneously cyclize to 4-hydroxy-1-methyl-2(1H)-quinolone. In 2020, Choi et al. (2020) constructed *E. coli* strains to produce 1,3-dihydroxy-9(10H)-acridone (DHA) and 1,3-dihydroxy-10-methylacridone (NMA) *de novo*. The pathway contains acridone synthase and N-methyltransferase (NMT) from *Ruta graveolens*, benzoate CoA ligase (badA) from *Rhodopseudomonas palustris* and anthranilate coenzyme A ligase (pqsA) from *Pseudomonas. aeruginosa.* Overexpression of shikimate pathway gene and an ACC from *Photorhabdus luminescens* optimized the production to 17.3 mg/L DHA and 26.0 mg/L NMA. Similar strategies were used to construct *E. coli* strains that can produce 4-hydroxycoumarin (4-HC), 2,4-dihydroxyquinoline (DHQ), and 4-hydroxy-1-methyl-2(1H)-quinolone (NMQ). Type III PKS acridone synthase from *R. graveolen*s, QNS from *Citrus macrocarpa* and the *P. aeruginosa* quinolone signal synthase pqsD were used in this study. With the use of strategies that increased the availability of chorismate, anthranilate, and malonyl-CoA, 255.4 mg/L 4-HC, 753.7 mg/L DHQ, and 17.5 mg/L NMQ were synthesized.

### Other type III PKSs

There are a variety of type III PKSs-derived polyketides, such as triacetic acid lactone, phloroglucinol, flaviolin, and aleosones, the starters of all of which are from acetyl-CoA or malonyl-CoA instead of shikimate pathway. In Figure 4 these starters are named simple CoA starters. Triacetic acid lactone is from the condensation of one acetyl-CoA starter and two malonyl-CoA extender units by the action of 2-pyrone synthase (2-PS). 2-PS from *Gerbera hybrida* (Gh2PS1) was identified and has been imported into *S. cerevisiae* (Cardenas and da Silva, 2014; Saunders et al., 2015), and *Y. lipolytica* (Markham et al., 2018; Yu et al., 2018) hosts to produce triacetic acid lactone. Numerous achievements that can biosynthesize triacetic acid lactone from glucose in microbial hosts have been accomplished. Till now, the highest production was achieved in *Y. lipolytica* (Markham et al., 2018). Bypassing pyruvate pathway and the overexpression of a β-oxidation-related enzyme PEX10 enhanced triacetic acid lactone production to 35.9 ± 3.9 g/L in bioreactor.

## Discussion

Type III PKSs are one of the most studied enzymes. This review has discussed the specific strategies and achievements of *de novo* plant polyketides biosynthesis in different microbial hosts, of which the biosynthetic pathways are almost fully elucidated. Other universal strategies to optimize the production of high value PNPs have been covered in a series of recent reviews (Li et al., 2018a; Cravens et al., 2019), including reconstructing biosynthetic pathways that are not fully elucidated, engineering cytochrome P450, or enhancing efficiency of multi-enzyme pathways.

The abilities to reconstruct and tune characterized plant polyketide biosynthetic pathways efficiently are important to the complete biosynthesis of valuable plant polyketides. High throughput and automation methods have the potential to transform current pace in increasing valuable plant polyketide synthesis in microbial hosts by generating and coupling modular genetic parts and modules in larger scale and faster speed, which have been highlighted by the development of multiplex automatic genome engineering (MAGE) (Kang et al., 2018) and various automation platforms (Radivojević et al., 2020). CRISPR/Cas is another powerful tool that can enable pathway construction in short time by assembling multiple genetic parts/genes simultaneously. For example, Gong et al. (2022) has developed a CRISPR/Cas mediated method customized for complicated plant pathway reconstruction in yeast, which has succeeded in reconstructing a 12-gene, *de novo* isoflavone pathway in one pot. Apel et al. (2017) developed a cloning-free toolkit for integration by employing CRISPR/Cas9 that can be applied to PNP synthesis too. These new genome editing methods are expected to advance the microbial biosynthesis of plant polyketides and other PNPs.
