# Corrigendum: Engineered biosynthesis of plant polyketides by type III polyketide synthases in microorganisms

**DOI:** 10.3389/fbioe.2022.1094116

**Published:** 2022-11-22

**Authors:** Chang Liu, Sijin Li

**Affiliations:** Robert F. Smith School of Chemical and Biomolecular Engineering, Cornell University, Ithaca, NY, United States

**Keywords:** type III polyketide synthases, plant polyketides, complete biosynthesis, microorganisms, biosynthesis strategies, biosynthesis achievements

In the published article, there was an error in Figure 1, Figure 2 and Figure 3 as published. The captions were mismatched but figures themselves were correct. The corrected Figure 1, Figure 2 and Figure 3 and their captions appear below.

**FIGURE 1:**
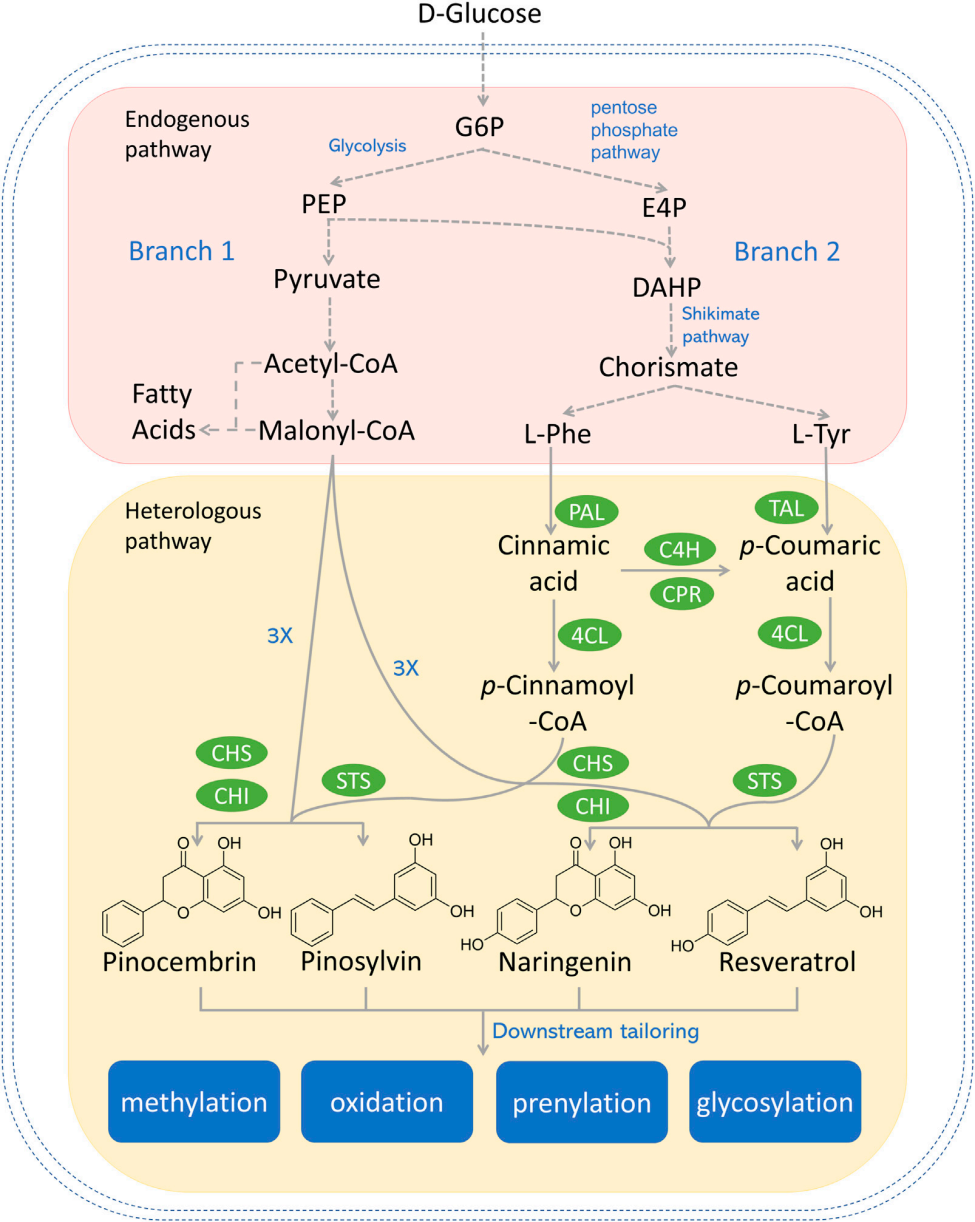
Reconstructed biosynthetic pathway for most explored type III PKS-derived polyketides (e.g., pinocembrin, pinosylvin, naringenin, and resveratrol) in microbial hosts. Dotted arrows refer to multiple steps. Genes and enzymes in green circle are heterologous genes from plants or bacterium. G6P, glucose-6-phosphate; PEP, phosphoenolpyruvate; E4P, erythrose-4-phosphate; DAHP, 3-deoxy-D-arabino-2-heptulosonic acid 7-phosphate; L-Phe, l-phenylalanine; L-Tyr, l-tyrosine; PAL, phenylalanine ammonia lyase; TAL, tyrosine ammonia lyase; C4H, cinnamic acid hydroxylase; CPR, P450 reductase; 4CL, 4-coumaroyl-coA ligase; CHS, chalcone synthase; CHI, chalcone isomerase; STS, stilbene synthase.

**FIGURE 2:**
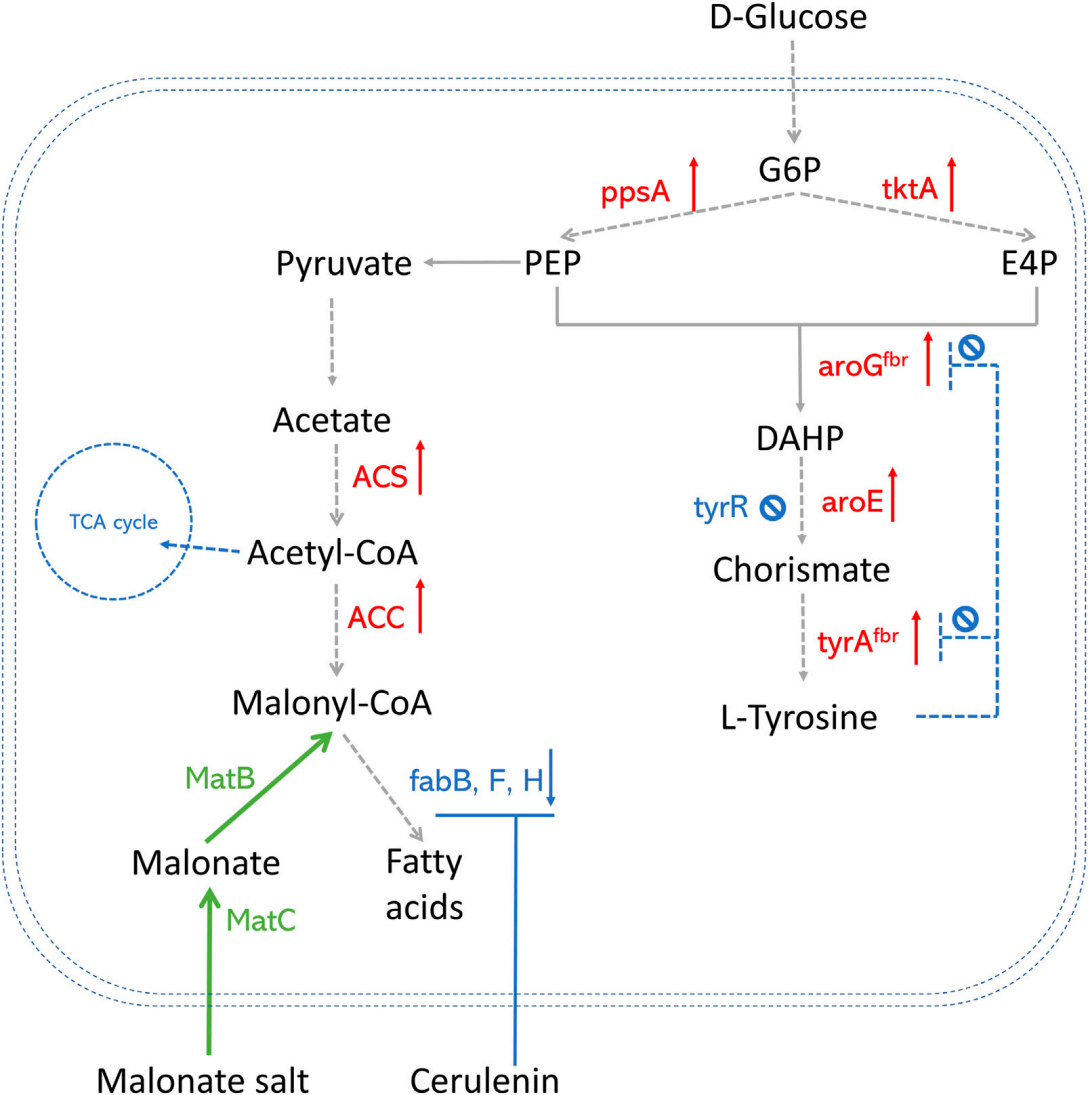
An overview of precursor enhancement engineering in *E. coli*. Genes and enzymes in red are overexpressed. Genes and enzymes in blue are deleted or downregulated. Genes and enzymes in green are heterologous expressed. Dotted arrows refer to multiple steps. G6P, glucose-6- phosphate; PEP, phosphoenolpyruvate; E4P, erythrose-4-phosphate; DAHP, 3-deoxy-D-arabino-2-heptulosonic acid 7-phosphate; fabH, gene that encodes 3-oxoacyl carrier protein synthase III; fabB/fabF, genes that encode the beta-ketoacyl-acp synthase I/II protein; MatB, malonylCoA synthetase; MatC, malonate carrier protein; ACS, acetyl-CoA synthase; ACC, acetyl-CoA carboxylase; TCA cycle, tricarboxylic acid cycle; ppsA, phosphoenolpyruvate synthase; tktA, transketolase; tyrAfbr, chorismate mutase-prephenate dehydrogenase feedback inhibition resistant variant; aroGfbr, DAHP synthase feedback inhibition resistant variant; TyrR, a DNA binding transcriptional regulatory protein.

**FIGURE 3:**
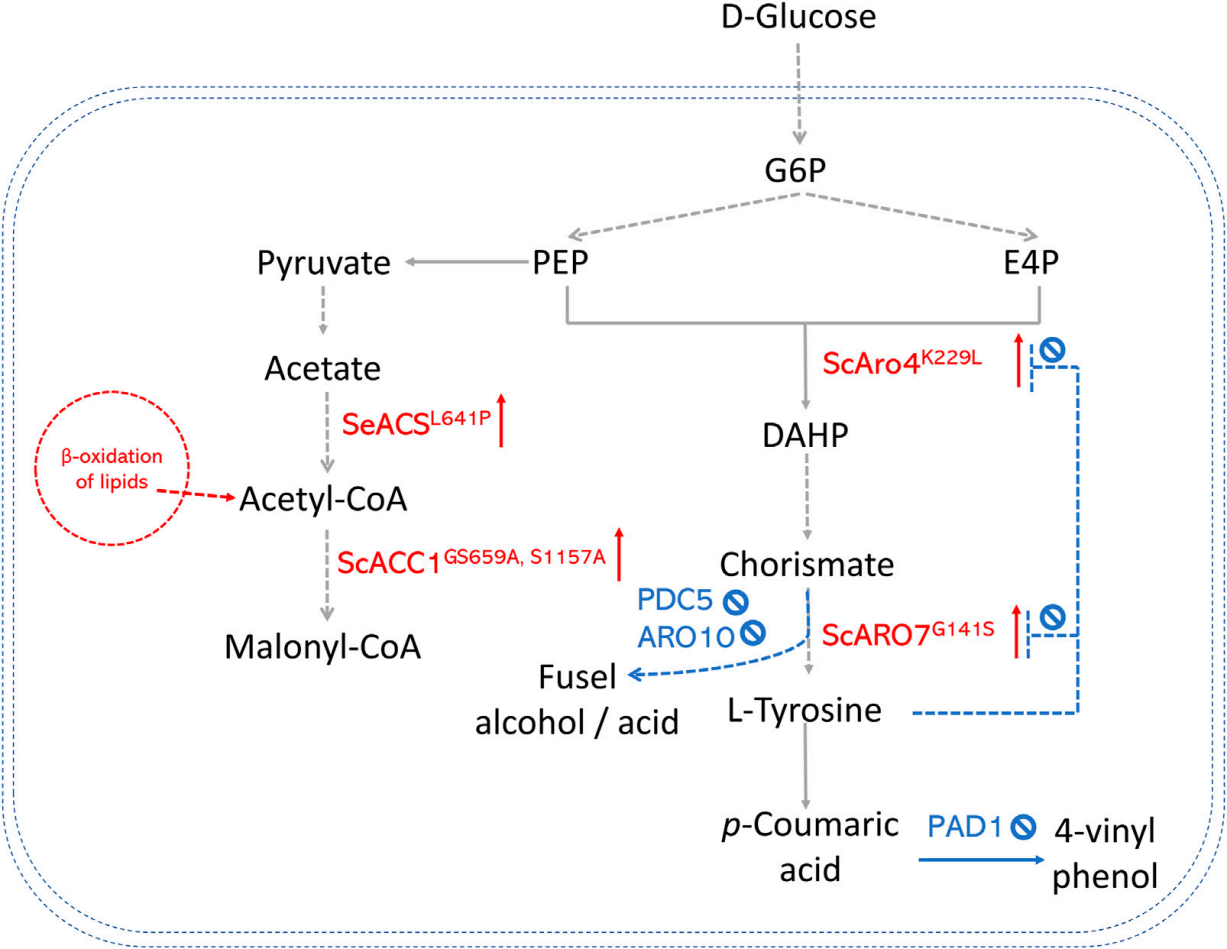
An overview of precursor enhancement engineering in *S. cerevisiae*. Genes and enzymes in red are overexpressed. Genes and enzymes in blue are deleted. Dotted arrows refer to multiple steps. G6P, glucose-6-phosphate; PEP, phosphoenolpyruvate; E4P, erythrose-4-phosphate; DAHP, 3- deoxy-D-arabino-2-heptulosonic acid 7-phosphate; ARO4K229L, DAHP synthase feedback inhibition resistant variants; ARO7G141S, chorismate mutase feedback inhibition resistant variants; PAD1, phenyl acrylic acid decarboxylase; PDC5, pyruvate decarboxylase; ARO10, phenylpyruvate decarboxylase; ACS, acetyl-CoA synthase; ACC, acetyl-CoA carboxylase.

The authors apologize for this error and state that this does not change the scientific conclusions of the article in any way. The original article has been updated.

## Publisher’s note

All claims expressed in this article are solely those of the authors and do not necessarily represent those of their affiliated organizations, or those of the publisher, the editors and the reviewers. Any product that may be evaluated in this article, or claim that may be made by its manufacturer, is not guaranteed or endorsed by the publisher.

